# Investigating the Accuracy of Wheelchair Push Counts Measured by Fitness Watches: A Systematic Review

**DOI:** 10.7759/cureus.45322

**Published:** 2023-09-15

**Authors:** Jonathan Byrne, Sarah Lynch, Arianne Shipp, Brandon Tran, Sukanya Mohan, Kelsey Reindel

**Affiliations:** 1 Dr. Kiran C. Patel College of Osteopathic Medicine, Nova Southeastern University, Clearwater, USA

**Keywords:** activity, smartwatch, push count, disability, wheelchair

## Abstract

Wheelchair users face an elevated risk of metabolic syndromes due to their sedentary lifestyles. One of the methods to prevent and treat various metabolic syndromes is regular physical activity, which varies among individuals based on their abilities. Monitoring physical activity among them can be performed by using wearable physical activity monitors (WPAMs), which utilize accelerometers and algorithms to track wheelchair push counts. However, the accuracy of push count detection varies among the devices due to technological limitations. The objective of this literature review was to evaluate the accuracy of WPAMs, specifically smartwatches, in measuring physical activity in the wheelchair population. This systematic literature review followed the Preferred Reporting Items for Systematic Reviews and Meta-Analyses (PRISMA) guidelines. The databases PubMed, Embase, and Cumulative Index to Nursing and Allied Health Literature (CINAHL) were searched in November 2022 for relevant articles. The initial search yielded 447 articles, seven of which were selected based on the inclusion criteria, which were as follows: participant ability to maneuver a wheelchair, arm- or wrist-worn WPAMs, and articles published after 2017. Among the devices studied, the Apple Watch was determined to be the most accurate calibration system for wheelchair users, with the lowest mean absolute percentage error (MAPE). Each succeeding generation of the Apple Watch (first to fourth) studied was more accurate than the previous. The review demonstrates that research on wheelchair fitness tracking remains scarce and further studies are required to address this issue.

## Introduction and background

According to the 2014 United States Census Bureau, 5.5 million adults over the age of 18 in the United States use a wheelchair for mobility [1]. Despite improvement in inclusion efforts over the years, people who use wheelchairs often end up excluded from societal participation due to poor infrastructure, stigma, and lack of adaptable equipment [2]. Additionally, wheelchair users are at a higher risk of developing metabolic syndromes as a result of sedentary lifestyles due to those barriers. However, regular physical activity (PA) has been shown to reduce the risk of developing diseases such as type 2 diabetes, obesity, and cardiovascular disease [3,4]. The Centers for Disease Control and Prevention (CDC) recommends that people with disabilities (PWD) should try to get at least 150 minutes of moderate-intensity aerobic physical activity per week based on their abilities [5].

One potential way to improve physical activity measurement is through the use of wearable physical activity monitors (WPAMs) [6]. Since 2016, WPAMS, such as Garmin, Apple Watch, and Fitbit, have grown in popularity with approximately one in five adults in the United States regularly using one as of 2020 [7,8]. WPAMs offer a convenient way to measure physical activity via daily step counting and exercise tracking; some also allow users to share their activity with others, allowing social support and competition that influence users’ motivation to exercise [9]. WPAMs can also be a valuable tool for clinicians, as they are a non-invasive option to monitor PA and rehabilitation efforts remotely and without relying on manual patient input [10]. Healthcare teams can monitor physiologic inputs in real-time and provide feedback to their patients on their progress [11]. However, traditionally, the features of WPAMs are built to measure step counts in able-bodied users, leaving wheelchair users with limited ability to use WPAMs to their full potential [10].

In the past, physical activity in the wheelchair-using population was difficult to assess using quantitative measures, and the information was limited to self-reported data [12]. However, with the rapid growth of WPAMs, there are currently various efficient tools to objectively measure everyday activity in this population, such as by using wheelchair push counts. There are many devices currently available in the market, including the Garmin VivoFit, Fitbit Flex, Jawbone UP24, and Apple Watch, that may be able to measure physical activity levels in wheelchair users. Since 2016, the most popular commercial smartwatch, the Apple Watch, has enabled wheelchair users to monitor daily push counts and other statistics for wheelchair physical activity [13]. 

Despite the availability of several brands, the accuracy of push count detection may vary among the WPAMs, and as technology rapidly improves, little is known about the current state of WPAMs and how precise they are in terms of measuring PA for wheelchair users. This can be a source of frustration for wheelchair users who wish to engage in fitness tracking. Some have resorted to improvising alternate methods to compensate for the lack of wheelchair-specific settings, which they then share with each other online [14]. According to some wheelchair users, wearables are currently not accurate for tracking steps and activities of daily living or informing them if their activity levels are sufficient. They have also noted other aspects of wearable technology not relevant to their experience in wheelchairs; namely features that suggest that the user should stand up and move [15].

In order to enhance the ability of wheelchair users to use WPAMs, improve their participation in society, and encourage physical activity, it is important to establish their validity for the unique needs of this population. In light of this, this systematic review aims to investigate the accuracy of various commercially available WPAM technologies, specifically watches, in assessing metrics of wheelchair push counts as a quantitative measure of physical activity in the wheelchair population.

This article was previously presented as a poster at the 2022 National SOMA Research Symposium on October 22, 2022; HCA NSU MD Research Day on November 4, 2022; and the 2022 World Disability and Rehabilitation Conference on November 12, 2022.

## Review

Methods

Data Sources and Literature Search

**Figure 1:**
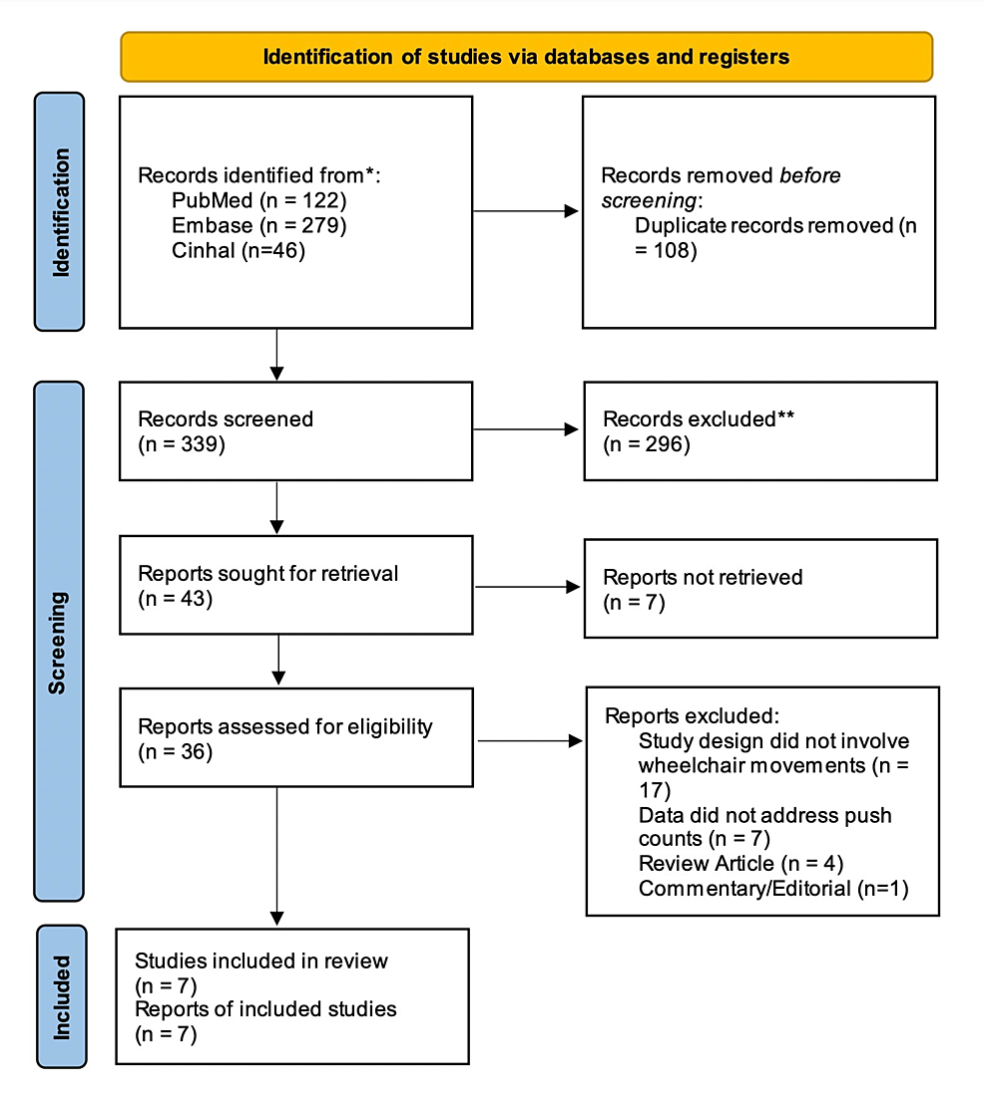
PRISMA flow chart depicting the selection process of the studies/articles PRISMA: Preferred Reporting Items for Systematic Reviews and Meta-Analyses

A literature search was done systematically and as per the most updated Preferred Reporting Items for Systematic Reviews and Meta-Analyses (PRISMA) [16] guidelines as of November 14th, 2022. The results of this systematic review are summarized in Figure 1. The databases that were searched included Medline/Pubmed, Embase, and Cumulative Index to Nursing and Allied Health Literature (CINAHL). The search was conducted in November 2022 using the following subject headings across all three databases: "((smartwatch) OR (Apple Watch) OR (Fitbit) OR (Fitness Tracker) OR (Body Worn Sensor)) AND ((disability) OR (Wheelchair))." A publication filter was used to restrict the search results. Only those papers that were published from January 1, 2017, to November 1, 2022, were included in this study to ensure that we had the most updated data regarding WPAMs and their accuracy for wheelchair users.

Study Selection Strategy

The initial search yielded 447 research articles across all three databases, which were analyzed using Rayyan software. After removing 108 duplicates, the remaining 339 articles were assessed and screened for titles and abstracts by two independent reviewers with regard to the appropriate patient population, study relevance, and study design in accordance with the inclusion and exclusion criteria. After this review, 43 studies were sought for full-length article retrieval; we could not retrieve the full versions of six articles, leaving 36 papers that were assessed for study population, data collection methods, and study design in accordance with the inclusion criteria. 

Only those articles with an experimental design were selected. Any review papers or editorial/commentaries were excluded from the final analysis. Only those studies that pertained to the use of commercially available wearable activity monitors and looked into the accuracy of push count data were chosen. Ultimately, seven articles that passed the final screening were included for full data extraction for this study. The inclusion and exclusion criteria are listed in Table 1. The final seven research articles were independently screened by one reviewer and were verified independently by a second reviewer for data extraction based on study methodology, design, and results. Table 2 summarizes the characteristics of each article selected [13,17-22]. If the second reviewer disagreed with any data extracted, both authors reached a consensus through a discussion. If no consensus was reached, a third reviewer was employed as the tiebreaker.

**Table 1:** Inclusion and exclusion criteria

Criteria	Description
Inclusion criteria	Participants used a wheelchair during the study, articles involving arm WPAMs, articles published after 2017, experimental validation studies, full-text available, validated proper data collection methods, published in English
Exclusion criteria	Articles published prior to 2017, articles involving non-wearable measuring devices, non-English articles

**Table 2:** Study characteristics

Study	Methodology	Study groups	Type of wearable
Benning et al., 2021 [22]	Original research (unspecified study type)	n=15 wheelchair users	Apple Watch Series 4 (WatchOS 6.2.6), Apple Watch Series 1 (data from Glasheen et al.)
Benning et al., 2020 [21]	Original research (unspecified study type)	n=20 able-bodied wheelchair users	Apple Watch Series 4 WatchOS 5.3.2 with iPhone 7, Fitbit Flex 2
Glasheen et al., 2021 [17]	Validation study	n=15 wheelchair users, n=15 able-bodies wheelchair users	Apple Watch Series 1 iOS 10.3.2 with iPhone SE iOS10.3.2
Glasheen et al., 2017 [18]	Validation study	n=4 wheelchair users, n=3 able-bodied wheelchair users	Apple Watch Series 1
Karinharju et al., 2021 [13]	Original research (unspecified study type)	n=26 wheelchair users (2 were excluded from final results, n=24)	Apple Watch Series 1, iOS version 10
Kressler et al., 2018 [19]	Original research (unspecified study type)	n=30 able-bodied wheelchair users	Garmin VivoFit, Fitbit Flex, Jawbone UP24
Leving et al., 2018 [20]	Original research (unspecified study type)	n=16 able-bodied wheelchair users	Activ8 Professional Activity Monitors (forearm and chair)

Data Extraction

A systematic review based on the extracted data was then performed on the validity of push count tracking while evaluating for differences across Apple Watches of different generations and other commercially available arm-wearable technology such as Garmin VivoFit, Fitbit Flex, Fitbit Flex 2, and Jawbone UP24. Studies were analyzed based on the following two categories: frequency of stroke patterns and wearable device technology.

Results

This systematic review summarizes the currently available research on the accuracy of detecting push counts among wheelchair users of current WPAMs in the commercial market. The review includes data collected from seven different types of wearable devices: Apple Watch Series 1, Apple Watch Series 4, Garmin VivoFit, Fitbit Flex, Fitbit Flex 2, Jawbone UP24, and the Activ8 Professional Activity Monitors. A comparative analysis is presented in Table 3. The wearable devices were all compared with the aid of video monitoring and/or manual counting. All participants in the seven selected studies were over the age of 18 years, physically able to maneuver a wheelchair whether disabled or able-bodied, intellectually capable of following commands, and included both males and females. Three out of the seven articles measured the difference in push counts based on the frequency of pushes [17,18,19]. The Activ8 was used primarily to differentiate push counts between various activities that a wheelchair user would do throughout an average day [20]. Four out of the seven articles involved direct comparisons between two or more wearable devices [18,19,21,22]. Overall, the calibrated Apple Watch, newer generation Apple Watches, and higher frequency pushes showed the most accurate push counts measured by wearable watches based on the data collected from the seven articles.

**Table 3:** Content analysis MAPE: mean absolute percentage error; MPE: mean percentage error; ICC: interclass correlation; p-value: probability value; CI: confidence interval; SOAD: sum of absolute differences, spm: strokes per minute, rpm: revolutions per minute, N/A: not accessible or not applicable

Study	MAPE or MPE	Push count, push count differences	P-value and CI	ICC	Comparison results, Bland Altman, t-test	SAOD	Sensitivity and positive predictive value	ρc
Benning et al., 2021 [22]	MAPE Apple Watch 4: 9.20%; difference in MAPE between Apple Watch 4 and 1: 11.42% (20.62% - 9.20%)	Apple Watch 4 direct observation avg.: 138.4 (86 - 271); mean push count difference between Apple Watch 4 and direct observation avg.: 12.33 (-3 - +38)	CI Apple Watch 4: 95%	ICC Apple Watch 4: 0.981	T-test for Apple Watch 4 vs. Apple Watch 1: t=3.011 (p = 0.008)	N/A	N/A	N/A
Benning et al., 2020 [21]	Apple Watch calibrated MAPE: 13.9%; Apple Watch uncalibrated MAPE: 22.8%; Flex 2 (drive A) MAPE: 148.4%	Push count difference between calibrated Apple Watch and examiner: +3 - +40; push count difference between uncalibrated Apple Watch and examiner: -20 - + 46; push count difference between Flex 2 and examiner: +105 - +184	N/A	ICC between subject and examiner: 0.981; ICC between drive A and B Flex 2: 0.785	N/A	Apple Watch calibrated SOAD: 271; Apple Watch uncalibrated SOAD: 401; Flex 2 (drive A) SOAD: 2,890	N/A	N/A
Glasheen et al., 2021 [17]	Apple Watch 1 treadmill MAPE for 30, 45, 60, variable spm respectively: 22%, 3%, 1%, 6%; Apple Watch 1 arm cycle ergometer MAPE for 45, 60, 80, variable rpm respectively: 1%, 1%, 1%, 4%; Apple Watch 1 obstacle course figure 8 MAPE for casual, fast, figure 8 respectively: 15%, 18%, 21%	N/A	N/A	Apple Watch 1 treadmill ICC for 30, 45, 60, variable spm respectively: -0.18, 0.47, 0.98, 0.22; Apple Watch 1 arm cycle ergometer ICC for 45, 60, 80, variable rpm respectively: 0.88, 0.95, 0.88, 0.97; Apple Watch 1 obstacle course figure 8 ICC for casual speed, fast speed, figure 8 respectively: 0.90, 0.79, 0.82	N/A	N/A	N/A	N/A
Glasheen et al., 2017 [18]	N/A	N/A	N/A	N/A	Apple Watch 1 Bland Altman with low stroke frequency mean difference: +/-60 strokes at 30 spm; Apple Watch 1 Bland Altman with higher frequencies (45 spm and 60 spm) mean difference: 2+/-8 and 1+/-3 strokes; Apple Watch 1 arm ergometry Bland Altman (45, 60, 80 rpm): 1+/-7, 2+/-3, and 2+/-4; Apple Watch 1 obstacle course Bland Altman: 3+/-15 strokes; Apple Watch 1 figure 8 Bland Altman: -15+/-40 strokes	N/A	N/A	Apple Watch 1 ρc 2 sided 95% CL Concordance (30, 45, 60 spm): -0.059, 0.348, 0.993; Apple Watch 1 arm ergometry ρc (45, 60, 80 rpm): 0.811, 0.954, 0.952; Apple Watch 1 obstacle course ρc: 0.755; Apple Watch 1 figure 8 ρc: 0.755
Karinharju et al., 2021 [13]	Apple Watch 1 MAPE = 13.5%	Apple Watch 1 push counts: 882 ± 239; Apple Watch 1 direct observation: 985 ± 300; Apple Watch 1 push counts mean difference = -103	Apple Watch 1 p<0.001; Apple Watch 1 Pearson correlation coefficient = 0.84 (95% CI)	Apple Watch 1 ICC = 0.77 (95% CI)	N/A	N/A	N/A	N/A
Kressler et al., 2018 [19]	MPE p<0.001 for increasing stroke frequency for all trackers; MPE for 30 spm roller: >46 for all trackers and declined to 3-6% at 60 spm; MPE for obstacle course: 12-17% for all trackers; MPE for arm ergometry Fitbit 60,80 rpm and Garmin 80 rpm with the best value at 1%	N/A	Roller wheelchair p-value for Garmin, Fitbit, and Jawbone respectively at random speeds: <0.001, <0.001, <0.001; ergometer p-value at 40, 60, 80 rpm for Garmin respectively: p = 0.094; p = 0.006; p = 0.477; ergometer p-value at 40, 60, 80 rpm for Fitbit respectively: p = 0.088; p = 0.031; p = 0.634; Ergometer p-value at 40, 60, 80 rpm for Jawbone respectively: p = 0.144; p = 0.164; p = 0.014	Roller wheelchair ICC for Garmin, Fitbit, Jawbone respectively at random speeds: 0.477, 0.640, 0.535; ergometer ICC at 40, 60, 80 rpm for Garmin respectively: 0.258, 0.499, -0.001; ergometer ICC at 40, 60, 80 rpm for Fitbit respectively: 0.265, 0.373, -0.078; ergometer ICC at 40, 60, 80 rpm for Jawbone respectively: 0.205, 0.187, 0.438	N/A	N/A	N/A	N/A
Leving et al., 2018 [20]	N/A	N/A	N/A	N/A	Relative time difference between Activ8 and video for 1 class: <10%; relative time difference between Activ8 and video for 2 classes of activities: 15.5%; relative time difference between Activ8 and video for 5 classes: <10%; overall agreement between Activ8 and video for 2 classes: 82.1% correctly divided into 2 classes; overall agreement between Activ8 and video for 5 classes: 56.5% correctly divided into 5 classes	N/A	2 class sensitivity and positive predictive value for Activ8: 77.7% and 78.2%; 5 class sensitivity and positive predictive value for Activ8: 52.8% and 51.9%	N/A

Comparing Wearable Devices

The findings illustrate that of the seven wearable devices that were used across the seven research articles we analyzed, the Apple Watch is the most accurate wearable technology to measure push counts. The Apple Watch calibrated the lowest mean absolute percentage error (MAPE) at 13.9% compared to the Apple Watch uncalibrated at 22.8% and Flex 2 at 148.4% [22]. The push count difference between the calibrated Apple Watch, uncalibrated Apple Watch, and Flex 2 was +3 to +40, -20 to +46, and +105 to +184, respectively [22]. Each successive generation of the Apple Watch has proven to be more accurate than the earlier generations (i.e., the Series 4 is more accurate than the Series 1) [21]. In the comparison between the Apple Watch 1 and Apple Watch 4, the Apple Watch 1 has a MAPE of 20.62%, and the Apple Watch 4 has a MAPE of 9.20% [21]. The Activ8 activity monitor is unique in that it is a combination of a monitor attached to the wrist and a monitor on the wheel [20]. Although it is comparable to the Apple Watch in accuracy, it is not commercially available, and it is considered a medical-grade device [20]. Furthermore, additional algorithmic changes are required for the Activ8 activity monitor to distinguish between different types of wheelchair activities [20]. The WPAM with the highest rate of errors and lowest intraclass correlation coefficient (ICC) included Fitbit, Jawbone, and Garmin [19]. The MPE for Fitbit Flex, Jawbone UP24, and GarminVivoFit for the obstacle course were all 12-17% [19]. Roller wheelchair ICC for the Garmin, FitBit Flex, and Jawbone respectively at random speeds was 0.477, 0.640, 0.535 [19]. However, the MPE decreased from 46% to 3-6% at a higher frequency of 60 spm for all devices [19].

Comparing Frequency and Stroke Patterns to Determine Accuracy

The aim of all seven of the studies was to see if existing WPAMs are accurate in detecting push counts. However, each of the studies employed different methodologies to reach these conclusions by using different values of frequency and stroke patterns. Three of the studies compared different frequencies of strokes and rotations at 30, 40, 45, 60, and 80 strokes per minute (spm) and rotations per minute (rpm) [17,21,22]. Of all the studies, one showed that the ICC is lower and the MAPE is higher for 30 spm versus 60 spm [18]. Using an arm cycle ergometer, the rpm showed a similar trend, providing evidence that higher frequencies will translate into more accurate push counts as measured by the Apple Watch 1 [13,18,19]. Using a figure 8-shaped obstacle course, the ICC decreased from 0.90 to 0.82, indicating that a wheelchair turn has decreased accuracy in measuring push counts measured through the Apple Watch Series 1 [13,17,18]. The figure 8-shaped obstacle course also shows a less accurate stroke count at 15+/-40 compared to the obstacle course at 3+/-15 strokes [13,17,18]. In other words, the Apple Watch Series 1 is more accurate in determining high-frequency push counts and less accurate in tracking push counts while turning [18]. Using an arm cycle ergometer, low-frequency push counts were measured to be +/-60 strokes at 30 spm compared to 1+/-3 strokes at 60 spm [22]. This data also shows that WPAMs are more accurate in high-frequency movements [17,18].

Activ8’s purpose is to measure the accuracy with regard to determining push counts for varying activities [20]. The Active8 was able to correctly divide two classes of activities with 82.1% accuracy. However, the overall agreement for five classes was 56.5%, meaning that Activ8 is able to differentiate between two activities, but the more activities, the less accurate it is in separating the type and number of pushes [20]. Overall, the 2-class sensitivity and positive predictive value for the Activ8 are 77.7% and 78.2%, respectively [20]. A turn of the wheelchair (such as in figure 8) and additional daily activities (such as playing basketball) in the wheelchair have lower accuracy push count measurements [13,19,20].

Discussion

Upon review of multiple WPAMs, we observed certain discrepancies in the accuracy of wearable technology for determining push count in wheelchair users. Consumer-level wrist-worn technologies tend to only be accurate in the detection of higher frequency movements, with the newer generations of Apple Watches most accurately capturing push count. The complexity and the amount of time taken to calibrate the WPAMs for wheelchair users seem to be significant drawbacks in terms of their day-to-day use. Another drawback for day-to-day use of WPAMs may be a higher initial financial investment when purchasing a fitness watch. 

The distinction between tracking step counts and wheelchair push patterns is another critical consideration when developing fitness-tracking technology for wheelchair users. While fitness watches measure step counts by recording arm swings correlated to heel strikes, the Apple Watch is unique because it identifies the main wheelchair push patterns and correlates them to the downward wrist angle that occurs during the wheelchair pushes. Wheelchair users typically use distinct hand pattern types when pushing a wheelchair, such as semicircular, arc push, and semi-loop over. The Apple Watch is able to differentiate these patterns in the accelerometer data to calculate caloric expenditure. The Apple Watch also includes accommodations for wheelchair users by replacing the stand ring with a roll ring in their fitness metrics and sending reminders to roll for a minute every hour [14].

For example, researchers tend to study the accuracy of push counts as a proxy for step counts; this leaves room for research on the accuracy of WPAMs in wheelchair users participating in other sports, such as basketball, tennis, or track, where different types of wheelchairs are used that require different types of strokes or amount of force used for each push [13,19,20]. A direction for future research could include how WPAMs measure exertion in wheelchair users depending on the effort required for each push and other factors such as the type/weight of the chair or the type of disability the user has. Similarly, the studies generally found that push count was most accurate for high-frequency pushing, meaning that they may be less accurate for other activities of daily living where the strokes may have lower or irregular frequency [13,17,19]. 

Additionally, results may vary based on the location in the body that the user wears their fitness tracker, a variable that may cause discrepancies due to the unique motions it takes to push a wheelchair. Some researchers used the watches on participants’ wrists, and others used them on the upper arms, chests, or wheels of the wheelchair. Future studies could aim to compare tracking accuracy based on the specific body location where users wear the device [15].

Finally, the latest version of the Apple Watch studied was a Series 4 released in 2018; newer versions may offer improved features [22]. However, there has been no published applicable data on wheelchair push count tracking since the release of the Apple Watch Series 4. Overall, the use of WPAMs to monitor the health and activity levels of wheelchair users has shown great potential in terms of accessibility and reliability, and this data has the potential to be used to formulate activity recommendations for wheelchair users. However, despite these advances, this review demonstrates that research on fitness tracking in wheelchair users remains limited given the low number of articles and applicable data sets that met this systematic review's inclusion criteria.

## Conclusions

Based on our results, the newer generation Apple Watch (among the devices analyzed in this review) was found to be the most accurate WPAM for measuring wheelchair push counts due to the wheelchair calibration system. However, in all the devices researched, higher-frequency pushes were found to have more accurate measurements compared to lower-frequency pushes. It can be concluded that the type of push and activity performed with the wheelchair has an effect on the accuracy of the WPAM in measuring push counts, as seen with multiple devices, primarily the Activ8. In the future, WPAM companies should continue to take into account the needs of wheelchair users in the research and development of their products. This strategic inclusivity aligns with the ongoing advancements in technology, presenting opportunities to foster a fitness environment that embraces inclusiveness for individuals of differing abilities.
